# Role of Surgery in Stages II and III Pediatric Abdominal Non-Hodgkin Lymphoma: A 5-Years Experience

**DOI:** 10.3390/cancers3021593

**Published:** 2011-03-29

**Authors:** Amany M. Ali, Heba A. Sayd, Hesham M. Hamza, Mohamed A. Salem

**Affiliations:** 1 Pediatric Oncology Department, South Egypt Cancer Institute, Assiut University, Asyut, Egypt; E-Mails: amanyamro@yahoo.com (A.M.A.); hebadina2007@yahoo.com (H.A.S.); 2 Surgical Oncology Department, South Egypt Cancer Institute, Assiut University, Asyut, Egypt; E-Mail: hesh_hamza@yahoo.com

**Keywords:** non-hodgkin lymphoma, pediatric, abdominal, surgery

## Abstract

Abdominal Non-Hodgkin lymphomas (NHL) are the most common extra nodal presentation of pediatric NHL. Our aim is to assess the role of surgery as a risk factor and to evaluate the impact of risk-adjusted systemic chemotherapy on survival of patients with stages II and III disease. This study included 35 pediatric patients with abdominal NHL treated over five years at South Egypt Cancer Institute (SECI), Assiut University, between January 2005 and January 2010. The data of every patient included: Age, sex, and presentation, staging work up to determine extent of the disease and the type of resection performed, histopathological examination, details of chemotherapy, disease free survival and overall survival. The study included 25 boys and 10 girls with a median age of six years (range: 2.5:15). Thirty patients (86%) presented with abdominal pain, 23 patients (66%) presented with abdominal mass and distention, 13 patients (34%) presented with weight loss, and intestinal obstruction occurred in six patients (17%). The ileo-cecal region and abdominal lymph nodes were the commonest sites (48.5%, 21% respectively). Burkitt's lymphoma was the most common histological type in 29 patients (83%). Ten (28.5%) stage II (group A) and 25 (71.5%) stage III (group B). Complete resections were performed in 10 (28.5%), debulking in 6 (17%) and imaging guided biopsy in 19 (54%). A11 patients received systemic chemotherapy. The median follow up duration was 63 months (range 51-78 months). The parameters that significantly affect the overall survival were stage at presentation complete resection for localized disease. In conclusion, the extent of disease at presentation is the most important prognostic factor in pediatric abdominal NHL. Surgery is restricted to defined situations such as; abdominal emergencies, diagnostic biopsy and total tumor extirpation in localized disease. Chemotherapy is the cornerstone in the management of pediatric abdominal NHL.

## 1. Introduction

Abdominal Non-Hodgkin's lymphomas (NHL) in pediatric patients are the most common extra nodal presentation of NHL, small and large intestines are the most frequent sites of involvement in the pediatric age group [1]. The prognosis for childhood abdominal NHL improved significantly with the realization that the majority of cases (85%) were disseminated from the start. The assignment of risk-adjusted systemic therapy as specific treatment protocols is necessary for long-term disease free survival [1,2]. The role of surgery in management of abdominal NHL has changed substantially so that surgical intervention has became restricted to defined situations such as; abdominal emergencies, diagnostic biopsy, total tumor extirpation and second look operations [3]. Aggressive operative debulking (defined as >90% tumor removal), prior to chemotherapy might delay institution of systemic therapy [3]. Patients' assignment to an appropriately intensive therapy according to their risk group is becoming the role with different protocols. Patients having minimal disease require less intensive therapy than do patients with extensive disease.

Our aim was to study the role of surgery as a dependent risk factor in management of abdominal lymphoma in pediatric patient with stages II and III disease and to analyze the impact of risk-adjusted systemic chemotherapy on survival of these stages.

## 2. Patients and Methods

This prospective study was carried out in the in South Egypt Cancer Institute, Assiut University, during the period from January 2005, to January 2010. The study was started after the agreement of the tumor board in SECI, Assiut University. Informed consent from the child's family was taken before including the child in the study.

### 2.1. Patients

The study included newly diagnosed non-disseminated pediatric abdominal NHL cases.

Patients were classified into two groups (group A and B) according to FAB (French, American, British) staging system for B-lymphoma, Table 1. Those who presented with minimal abdominal disease that is completely resected were classified as group (A), and patients with extensive non-resectable abdominal mass as group (B). We excluded disseminated cases (group C according to the FAB staging system), who require very intensive chemotherapy and supportive-care.

### 2.2. Staging Work-up and Classification

The presenting symptoms and extent of disease were determined by history, physical examination, and baseline complete blood picture, liver function tests, kidney function tests, lactate dehydrogenase (LDH) and uric acid as a tumor bulk indicator. Bone marrow aspiration/biopsy, CSF cytology, chest XR, abdominal ultrasound and CT scan of the abdomen were performed.

After staging work up, histopathologic diagnosis from the presenting mass was done through surgical resection or imaging guided biopsy. Patients were candidates for complete surgical resection if they had localized disease. In patients with non-resectable disease, initial surgery included the least invasive procedure to relieve the emergent presentation or biopsy to establish the diagnosis.

Resection was defined as complete if all gross tumors were excised or incomplete if gross tumor was left *in situ* and those undergoing biopsy only.

All pathologic specimens were reviewed and classified according to new WHO-Classification system. Biopsy specimens were subjected to immunohistopathological examination when there was doubt in confirmation of the pathology.

### 2.3. Chemotherapy

After establishment of the exact pathology and the stage of disease, and as soon as the patient's general condition allowed, assignment of risk-adjusted systemic chemotherapy was started. Those who presented with bulky tumor size after incomplete resection or biopsy only were candidates for a cytoreductive prephase in the form of fractionated “COP” (oncovine, prednisolone and fractionated cyclophosphamide). Further chemotherapy was according to risk group:

*Group A:* Patients of this group were treated by six cycles of COP AD (Cyclophosphamide, Oncovine, Doxorubicin and Prednisone), Table 2. Cycle every 21 days.
*Group B:* In this group we used the Modified LMB 89 Protocol, Table 3, which is convenient with our resources for supportive care. The general scheme of the LMB protocol includes cytoreductive phase (fractionated COP) given one week before the intensive induction (two cycles of COP ADM), to be followed by a consolidation phase (2 cycles of CYVE) and 4 cycles of maintenance. Modification was done on the dose of methotrexate; we changed it from 3 g/m^2^ to 1 g/m^2^. Methotrexate was administered over 6 hours with folinic acid rescue (15 mg/m^2^ every 6 hours × 6 doses), which was given 24 hours after the start of methotrexate. Another modification involved the doxorubicin. We changed the dose from 60 mg/m^2^ into 40 mg/m^2^. The intrathecal injection was adjusted according to the age (9 doses). Intervals between courses were to be as short as possible and chemotherapy was to be started as soon as the absolute neutrophil count attained more than (500/μL) and platelets number more than (50,000/μL).

### 2.4. Statistical Methods

The study cut off limit was January 2010. Disease Free Survival (DFS) was calculated from the first day of diagnosis to the last date of follow up. DFS was estimated with the Kaplan-Meier method. Differences were considered significant when P ≤ 0.05.

## 3. Results

The study included 35 children collected over five years at the Pediatric Department of South Egypt Cancer Institute, Assiut University. They were 25 boys and 10 girls with a median age of six years (range: 2.5: 15 years). The most common pathology was Burkitt's lymphoma which was diagnosed in 29 cases (83%) and large B-cell lymphoma in six cases (17%).

The most common symptom was abdominal pain which was present in 30 patients (86%), followed by abdominal swelling which was present in 23 patients (66%), 13 patients (37%) presented with weight loss and six patients (17%) presented as emergency intestinal obstruction, Table 4.

The laboratory characteristics of the patients are shown in Table 4; 10/25 patients (40%) had profound anemia (Hemoglobin level below 7 g/dL) all from group B, 11/35 patients (31.5%) had hypo-albuminemia. 2/25 patients (8%) of group B had renal function impairment, serum creatinine raised above 2-fold of normal. Twenty-two (63%) had LDH levels below 1000 U/L while 13 patients (37%) had their LDH level above 1000 U/L, all from group B. The median LDH level was 430 U/L (range 120–3000).

Table 5 shows the surgical events in this work. Nineteen patients (54%) had bulky abdominal disease and were diagnosed by CT guided biopsy. Sixteen (46%) patients were submitted to abdominal exploration. Complete resections were done in 10/16 (62.5%), and incisional biopsy in six patients (37.5%).

In the 10 patients who had complete resection; right hemi-colectomy was performed in eight patients (50%) and small intestinal resections and anastomosis was performed in two patients (12.5%). There were no intra-operative complications, and all of the patients had smooth post-operative recovery except for one who presented with small intestinal obstruction. He had wound dehiscence after resection and anastomosis procedure. The hospital stay in the surgical ward ranged from 5 to 7 days (according to the procedure performed) and it was prolonged for 19 days in the complicated case. There was no post-operative mortality or delayed chemotherapy administration except for the complicated case.

Twenty-nine patients had histopathology results of Burkett's lymphoma and six patients had large cell type lymphoma. The immuno-histopathology was performed for confirmation in 12/35 patients (34%) and all had the B-cell phenotype.

According to the FAB staging system for B-Cell lymphoma, 10 out of 16patients had small volume disease that had complete resection, classified as group A. The six patients whom had incisional biopsy were gathered with the 19 patients who had CT diagnosed bulky disease and were classified as group B. Risk adjusted systemic therapy was assigned according to the protocols in Table 2 and Table 3.

### 3.1. Therapy Results

Ten patients were treated in group A, 25 patients in group B .Thirty patients (86%) are alive and on regular follow up, nine from group A and 21 from group B. Three patients (9%) died during the induction therapy and two patients (6%) after relapse.

### 3.2. Response to Pre-phase Treatment

Response was evaluated in 25 group B patients. Among the 25 patients, 18 patients (72%) had good response. Seven patients (28%) had poor response.

### 3.3. Remission

A total of 30 patients (86%) continue in CR, nine patients (90%) from group A and 21 (84%) patients from group B.

### 3.4. Relapses

Two patients (6%) relapsed , one from group A and the other from group B .The first patient had abdominal mass (group A stage II), received six cycles of COPAD and after two months from follow up the patient developed local relapse and the patient received aggressive chemotherapy, but the patient died due to progression of the disease. The second patient had abdominal mass (group B stage III). He received two cycles of chemotherapy and achieved partial remission. The patient received another two cycles of chemotherapy with no response, and. the patient developed CNS relapse and died from pulmonary edema.

### 3.4. Toxicity Related Deaths

Five patients (15%) died during therapy, one patient from group A died after relapse. Another four patients from group Bdied, three during the induction course and one patient after relapse. Mortalities were directly related to the high dose methotrexate which lead to severe myelo-suppression, mucositis, gastroenteritis and. septicemia

### 3.5. Treatment-Related Toxicity and Morbidity

All morbidities occurred in group B. Myelo-suppression was the main treatment complication, especially during the COP ADM and CYVE courses. More than 80% of the patients experienced febrile neutopenia requiring intravenous antibiotics and blood transfusion. Mucositis was the second main complication (72%), especially in the COPADM courses due to the combination of methotrexate and doxorubicin. Other main complications were; severe gastroenteritis and dehydration (32%), toxic hepatitis (28%), sever infection and septicemia (9%).

### 3.6. Survival Analysis

**Figure 1.**
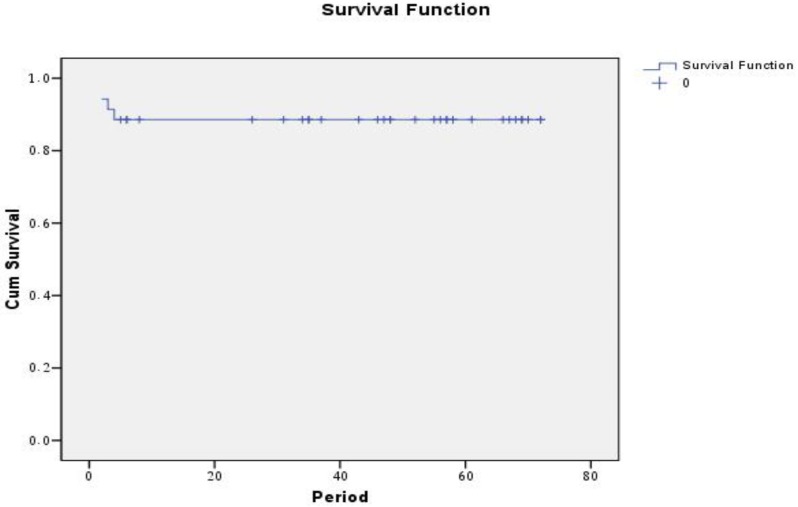
Disease free survival rates (DFS) for all patients.

**Figure 2.**
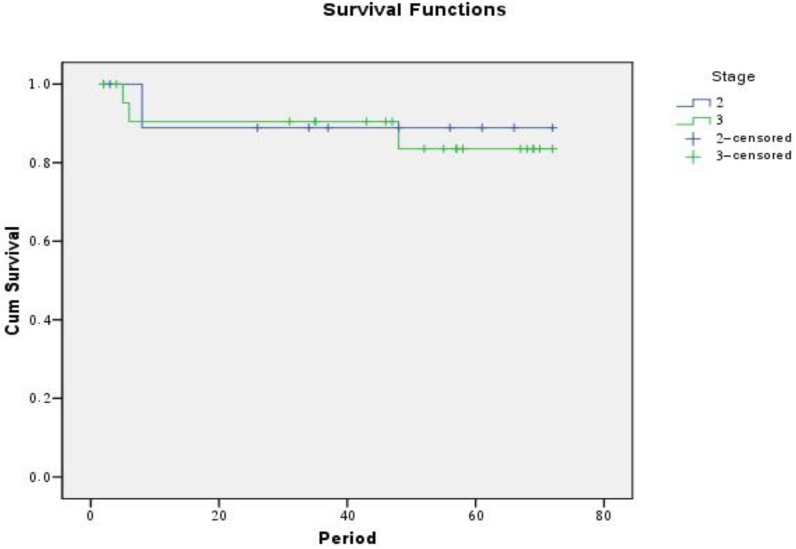
Disease free survival rates (DFS) of group A *versus* group B.

The median follow up duration was 51 months (range 41-62 months). The five year disease free survival rates (DFS) were 88.2% for all patients, 90% for group A and 87.5 for B (Figure 1 and Figure 2).

**Figure 3.**
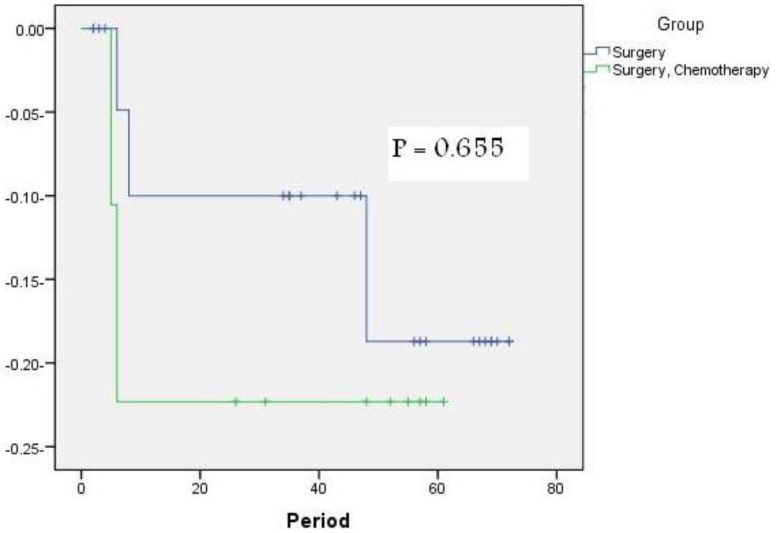
Complete resection with insignificant P value.

Figure 3 shows that the extent of resection did not have a significant influence on EFS with a P value (0.655) and cannot be interpreted as an independent predictor.

## 4. Discussion

This study is a trial to evaluate the effect of surgery on the survival of pediatric patients presented with non-metastatic abdominal non-Hodgkin's lymphoma in south Egypt community. We analyzed several prognostic factors in regard to their influence on the event free survival. Factors included were site distribution, age, sex, presentation (emergency or not), stage at the time of diagnosis and extent of resection.

The distribution of our cases fits with this fact as 25 of our 35 patients (71.5%) had their lesions in the bowel. Two of the patients had their lesions in the small intestine (6%), in 17 patients (48.5%) the ileo-caecal region was the site of involvement and in six patients (17%) large intestine was involved. Similar result was reported in other studies [3-5].

The peak age for abdominal NHL in children is 5-15 years. In our series, the median age for our patients was six years, which is similar to what was reported by Khairy *et al* [6], while it was less than the median age of patients reported by Ebeid *et al* [2] of eight years.

As regards sex, there is a marked predominance of males in all series of childhood NHL [1]. In the present study the male to female ratio was 2.3: 1 which is lower than the ratio reported by Ebeid *et al* [5] and close to what was reported by Cairo *et al* [7].

Patients with sporadic Burkitt's lymphoma primarily manifested in the abdomen with picture-like appendicitis (ileocecal area), intussusception, abdominal pain, change in bowel habits, GIT bleeding and perforation. Around 25% of such patients may come with a right iliac fossa mass [3]. Our patients in this study presented with all the above mentioned presentations and in six patients the presentation was intestinal obstruction. This is similar to reports in other series [1,2,7-9].

According to the WHO histological classification of NHL in children, B-cell immuno-phenotypes (Burkitt, Burkitt-like, large B-cell) most commonly present in the abdomen as the primary site. Burkitt's lymphoma is by far the most common subtype [10] . This fact matches with our results where the incidence of Burkitt's lymphoma in our study cases was 83%, which is also in agreement with other reports [1,7,8].

Childhood NHL is a highly aggressive disease which grows rapidly and disseminates early. The majority of patients (70%) presented with advanced disease at diagnosis [8,9]. In the current study, 25 of patients (71.5%) were stage III and 10 patients (28.5%) were stage II. This distribution is similar to other reported series where stages II and III usually represent around 75% of cases [3,4].

The best reported results indicate that approximately 90% of all patients can be cured when treated optimally. The majority of patients in the developing countries can be cured in centers in which the cost of the drugs is met and there is reasonable supportive care [1,2,7].

Risk group assignment; although following similar principles, differs in different cooperative groups. In general, patients with B-cell tumors in which all disease has been surgically removed are considered to be in the lowest risk group. Patients with CNS and /or BM infiltration are considered to be in the highest risk group [2,7]. In our study we classify our patients into two groups, patients with complete surgical resection as group A and other non disseminated un-resectable as group(B), which is in agreement with others [1,2,11].

Surgery can be beneficial in a variety of instances in the overall treatment of pediatric NHL including; biopsy for initial diagnosis, exploratory laporatomy in patients with acute abdominal disease, and surgical resection during acute emergencies [12,13].

On analyzing the survival results of this study, data cannot support the influence of surgery as independent predictor of survival in non-metastatic disease, and the only independent risk factor was the stage at the time of diagnosis. This can be explained by that tumor extirpation, determined by the stage of disease, the effect of the extent of resection as an independent prognostic factor cannot be interpreted separately. This is consistent with other studies [2,5,12,13]. However we recommend total extirpation for localized disease if not mutilating, to minimize tumor burden and to avoid intensified chemotherapy.

Because children with partially resected or biopsied tumors are treated identically, partial resections neither add to survival nor prevent the patients from entering the higher risk group. Thus, in patients with locally advanced disease where laparotomy is needed for diagnosis, surgery should be limited only to the least invasive procedure, such as biopsy or a simple resection-anastomosis to relieve the emergency situation. So, we could avoid any major surgical procedure that may lead to higher incidence of postoperative complications and more importantly may lead to delayed initiation of chemotherapy. This conclusion is essentially attributed to the reported excellent results achieved by chemotherapy in the cases of biopsied or partially resected pediatric lymphoma [12-14].

The results of several new protocols in which the principle of risk stratification is used, demonstrate better results with overall disease free survival approximates 90%. Patients with stage II has 98-100% disease free survival rate *versus* 76-86% in those with most extensive disease [12,14]. At five years, DFS for 10 of our patients in group A was 90%, this is in agreement with the current results of international studies of group A [1,15].

At five years, DFS for 25 patients of group B on the modified LMB 89 protocol was 87.5%. This is in agreement with the current results of international studies of group B where the five year EFS in the SFOP trail was 92%[10,16,17], and in the POG trail was 83% [7]. Our results also was in agreement with most Egyptian studies [5,6] and developing countries as in Malawi and Ghana [18,19].

A relapse indicates an extremely poor prognosis regardless of the site of relapse, tumor histology, other original prognostic factors, prior therapy, or time from diagnosis to relapse .For this reason, the selection of the most effective front- line treatment is critical [5,8]. In BL recurrent disease has become rare and is almost never observed beyond one year after the initiation of therapy [2].

Two (6%) of patients relapsed; the low relapse rate is in agreement with results obtained from the other series [6,18,19]. The high survival rate in our study was due to better stratification of the patients, low LDH level, good response to pre-phase fractionated COP and satisfactory supportive care.

## 5. Conclusions

The extent of the disease at presentation is the most important prognostic factor in patients with pediatric abdominal lymphoma. Surgery still plays an important role in situations such as complete resection in localized disease, management of emergency presentation, diagnostic biopsy and there is no value of debulking. Chemotherapy represents a cornerstone in the treatment of these patients and offers the patients an excellent chance for long-term disease free survival.

## Figures and Tables

**Table 1.** FAB* Staging System for B-Cell Lymphoma.

**Group A:** Completely resected stage 1 (St. Jude staging system)
Completely resected abdominal stage II (St. Jude staging system)
**Group B:** All patients not eligible for group A or C
**Group C:** Any CNS involvement† and/or bone marrow involvement (≥25% blast)

* *FAB:* French, American, British;

† CNS: any L3 blast, cranial nerve palsy or compression, intracerebral mass, and/or parameningeal compression.

**Table 2.** COP AD (Cyclophosphamide, Oncovine, Doxorubicin and Prednisone) Protocol for Localized Non Hodgkin Lymphoma for Group A.

**Drug**	**Dose/Route**	**Day(s)**
Cyclophosphamide	1000 mg/m2 IV	Dose divided into 4 doses every 12 hours
Adriamycine	40 mg/m2 IV	Dl
Oncovine	1.4 mg/m2 IV	Dl
Prednisone	60 mg/m2per day PO	Dl-5
CYCLE: 21 days 6 CYCLES		

**Table 3.** Modified LMB 89 Protocol for Group B.

Reduction phase: Fractionated COP		
Induction: 2 cycles started atd8		
COPADM 1		
Oncovine	2 mg/m2 IV	Dl
Methotrexate (high dose)	1 g/m2 IV	Dl over 6 h infusions.
Folinic acid	15 mg/m2PO	q6h x6doses D 2-4
Intrathecal	Age adjusted	D 2,4 and 6
Cyclophosamide.	500 mg/m2 IV	D2-4
Adriamycin	40 mg/m2 IV	D2
Prednisone	60 mg/m2 PO	DI-5
COPADM 2 as COPADM 1 expect Oncovine D1.D5		
CONSOLIDATION: 2 cycles of CYVE		
Cytarabin HD	1 g/m2/3h IV	Dl-4
Vipiside	200 mg/m2 IV	Dl-4
Maintenance 4 cycles every 21 days		
Cycle 1		
Prednisone.	60 mg/m2 PO	DI-5
Mtheotrexate (high dose)	1 g/m2 (over 6h.infusion) IV	Dl
Folinic acid	15 mg/m2IV12dosesIV	D2-4
Intrathecal	Age adjusted	Dl
Cyciophosamide	500 mg/m2 IV	D2,3
Adriamycin	40 mg/m2 IV	D3
Oncovine	2 mg/m2 IV	Dl
Cycle 2		
Cytarabine	100 mg/m2 /d SC	DI-5 in 2 injections.
Vepiside	150 mg/m2 /d IV	Dl-3
Cycle 3 as cycle 1 without high dose mthotrexate and Cycle 4 as cycle 2		

**Table 4.** Clinical Characteristics of 35 Abdominal Non-Hodgkin lymphomas (NHL) Patients.

**Patients Characteristics**	**All Patients/%**	**Group A** (n = 10)	**Group B (n = 25>)**

**Age**			
Age Range	2.5-15y.	5-7y.	3-15y.
Mean ± S.D.	6.89 y. ±4.96	8.1±3.088	5.2±2.569
Median	6y.	6y.	6y.

**Gender**			
Male	25(71.5%)	8	17
Female	10 (28.5%)	2	8

**Clinical Presentation**			

Abdominal Pain	30(85.7%)	11	19

Abdominal Swelling	23 (66%)	7	16

Vomiting	15 (42.8%)	9	6

Constipation	2 (6%)	2	0

Intestinal obstruction	6 (17%)	6	0

Appendicitis like pain	5(14%)	5	0

Weight loss	13(37.1%)	0	13

Fever	9 (27%)	0	9

**Laboratory characteristics**			
Profound anemia	10/25 (40%)	0	10
Hypoalbuminemia	11/35(31.4%)	0	11
Impaired renal function	2/25 (8%)	0	2
			
***Elevated LDH.***	35	10(28.5%)	25(71.4%)
<1000 u\L	22 (62.8%)	10(28.5%)	12 (34.2%)
			
>1000 u\L	13(37.1%)	0	13(37.1%)

**Table 5.** Surgical Management of 35 abdominal NHL.

	**All Patients**	**Group A**	**Group B**

**Site of involvement**			
Large bowel Small bowel	6(17%) 2(6%)	0	6
		0	2
Ileo-cecal	17 (48.5%)		7
		10	3
Retroperitoneal region (Abdominal lymph nodes)	3 (9%) 7(21%)	0	7
		0	

**Number of explored patients**	16		
Complete resection.	10/16(62.5%)	10	0
Rt. Hemicolectomy	-	8	0 0
Bowel resection and anastomosis	-	2	
Incision biopsy	6/16(37.5%)	0	6

**Imaging guided biopsy 19 patients.**	19/35 (54%)	0	19
